# Retrospective study on the usefulness of pulse oximetry for the identification of young children with severe illnesses and severe pneumonia in a rural outpatient clinic of Papua New Guinea

**DOI:** 10.1371/journal.pone.0213937

**Published:** 2019-04-15

**Authors:** Julien Blanc, Isabella Locatelli, Patricia Rarau, Ivo Mueller, Blaise Genton, Noémie Boillat-Blanco, Mario Gehri, Nicolas Senn

**Affiliations:** 1 Department of Ambulatory Care and Community Medicine, University of Lausanne, Lausanne, Switzerland; 2 Vector Borne Disease Unit, PNG Institute of Medical Research, Madang (MAD), Papua New Guinea; 3 Population Health & Immunity Division, Walter & Eliza Hall Institute of Medical Research, Melbourne, Australia; 4 Barcelona Institute of Global Health (ISGLOBAL), Barcelona, Spain; 5 Malaria, Parasites & Hosts Unit, Institut Pasteur, Paris, France; 6 Infectious Diseases Service, University Hospital, Lausanne, Switzerland; 7 Swiss Tropical and Public Health Institute, University of Lausanne, Switzerland; 8 Children’s Hospital, Lausanne, Switzerland; University of Bern, SWITZERLAND

## Abstract

**Objective:**

This secondary analysis of data of a randomized controlled trial (RCT) retrospectively investigated the performance of pulse oximetry in identifying children with severe illnesses, with and without respiratory signs/symptoms, in a cohort of children followed for morbid episodes in an intervention trial assessing the efficacy of Intermittent Preventive Treatment for malaria in infants (IPTi) in Papua New Guinea (PNG) from June 2006 to May 2010.

**Setting:**

The IPTi study was conducted in a paediatric population visiting two health centres on the north coast of PNG in the Mugil area of the Sumkar District.

**Participants:**

A total of 669 children visited the clinic and a total of 1921 illness episodes were recorded. Inclusion criteria were: age between 3 and 27 months, full clinical record (signs/symptoms) and pulse oximetry used systematically to assess sick children at all visits. Children were excluded if they visited the clinic in the previous 14 days.

**Outcomes:**

The outcome measures were severe illness, severe pneumonia, pneumonia, defined by the Integrated Management of Childhood Illness (IMCI) definitions, and hospitalization.

**Results:**

Out of 1921 illness episodes, 1663 fulfilled the inclusion criteria. A total of 139 severe illnesses were identified, of which 93 were severe pneumonia. The ROC curves of pulse oximetry (continuous variable) showed an AUC of 0.63, 0.68 and 0.65 for prediction of severe illness, severe pneumonia and hospitalization, respectively. Pulse oximetry allowed better discrimination between severe and non-severe illness, severe and non-severe pneumonia, admitted and non-admitted patients, in children ≤12-months of age relative to older patients. For the threshold of peripheral arterial oxygen saturation ≤ 94% measured by pulse oximetry (SpO_2_), unadjusted odds ratios for severe illness, severe pneumonia and hospitalization were 6.1 (95% Confidence Interval (CI) 3.9–9.8), 8.5 (4.9–14.6) and 5.9 (3.4–10.3), respectively.

**Conclusion:**

Pulse oximetry was helpful in identifying children with severe illness in outpatient facilities in PNG. A SpO_2_ of 94% seems the most discriminative threshold. Considering its affordability and ease of use, pulse oximetry could be a valuable additional tool assisting the decision to admit for treatment.

## Introduction

Pneumonia is the leading infectious cause of mortality in children under five years, responsible for 704 000 deaths in 2015 [1]. The majority of deaths due to pneumonia occur in developing countries as it is strongly related to socio-economic factors such as malnutrition, poverty or inadequate access to the health system [2].

Hypoxemia is one of the major complications of pneumonia, associated with severe pneumonia [3]. It is also strongly associated with mortality; children with hypoxemia were more than four times more likely to die in the next five days compared to children without hypoxemia in a Kenyan study [4].

In developed countries, hypoxemia is usually identified using pulse oximetry, whereas in resource-poor settings indirect clinical signs such as cyanosis, or others signs reflecting respiratory distress (high respiratory rate, lower chest wall indrawing, head nodding, nasal flaring, grunting, drowsiness, and/or inability to drink/breastfeed) are usually used. The latter signs are correlated with a higher risk of mortality than specifically with hypoxemia. Several studies evaluated the association between hypoxemia and signs and symptoms. These are recognized as useful to evaluate children presenting with Acute Respiratory Infections (ARIs) in settings without access to pulse oximetry, but most of the studies highlighted limited predictive values with high variability in clinical performance and inter-observer variability between studies [5–11]. In a Papua New Guinean prospective observational study, cyanosis was highly specific but poorly sensitive to identify children with hypoxemia, moreover it is a late sign and shows considerable inter-observer variability, failing to detect hypoxemia in 30–40% of the cases [12]. Finally, a meta-analysis by Zhang et al. (2011) concluded that signs with high sensitivity usually lack specificity and that neither single nor combined signs/symptoms have enough performance to reliably predict hypoxemia in children with ARIs [13].

Few studies have investigated the use of pulse oximetry to detect hypoxemic diseases in children in resource-poor countries. A Malawian study showed that implementation of routine pulse oximetry increases the referral of severely hypoxemic children with pneumonia and that following the WHO guidelines, care-givers are likely to under-refer children with severe hypoxemia [14]. Two studies showed that models combining pulse oximetry-derived information with clinical and paraclinical information predicted the need to hospitalized children consulting an emergency department and outpatient facility in Bangladesh [15,16]. A study conducted in Peru in the ‘90s in a large paediatric emergency department found that pulse oximetry could help in assessing severity of the disease and facilitated referral of patients requiring oxygen to the appropriate centers [17].

The present observational study investigated the performance of pulse oximetry to identify children with severe illnesses with- and without respiratory signs/symptoms among a cohort of children followed for morbid episodes in an intervention trial assessing the efficacy of Intermittent Preventive Treatment for malaria in infants (IPTi) in Papua New Guinea (PNG).

## Methods

### Study design

This is a secondary analysis of the randomized controlled trial (RCT) data from a cohort of children aged 3–27 months followed-up as part of a double-blinded RCT on the prevention of malaria (IPTi) [18].

### Study sites

The IPTi trial was conducted on 1121 children aged 3–27 months from June 2006 to May 2010 (www.clinicalTrials.gov: NCT00285662) [18]. The study took place on the north coast of PNG in the Mugil area of the Sumkar District, Madang Province. It included 20 villages situated in an ~400-km2 coastal area 30–60 km north of Madang town. Two main health centers serve this region, Mugil and Alexishafen, complemented by several first-aid posts. The health centers provide basic in-patient care (including deliveries) and run a daily outpatient clinic for common acute medical problems. Nurses run the centers and patients (adults or children) need to be referred to Madang hospital to see a doctor (60 km away). First-aid posts are rudimentary and remote health facilities in presence of a nurse living in the community. Pulse oximetry was implemented and used systematically to assess sick children in the Mugil site from December 2007; therefore, only data collected after this date and from this site are used in the present study.

The study was carried out in accordance with Good Clinical Practice (ICH GCP E6) guidelines and externally monitored by two independent monitors and the Data Safety Monitoring Board. The study was approved by the PNG Medical Research Advisory Committee (MRAC number 05.20). The trial was registered on http://www.clinicaltrials.gov (number NCT00285662) and formed part of the IPTi Consortium (http://www.ipti-malaria.org) [18].

### Study population

The study was conducted on a pediatric population (669 children aged 3 to 27 months) enrolled in the IPTi trial and visiting the study health facilities in Mugil for any health-related problem.

### Morbidity surveillance

A passive case detection system was maintained at the study health-center clinic of Mugil to ensure continuous morbidity surveillance of study participants. Children visited the health center at routine vaccination time points (3, 6 and 9 months) and parents of participating children were asked to visit the clinic free-of-charge whenever their child was sick. They were seen by one of the study nurses, often accompanied by a health-center staff member. Community reporters were responsible for reporting any deaths that occurred. Additionally, when a child did not show up for a regular follow-up visit, study staff enquired to see what had happened to the child. Death rarely occurred in the health facilities. Finally, at the end of the study, a last check was performed to ensure that children were still living (part of the main study protocol). The passive surveillance records collected were used to perform the present analysis.

The IPTi study was explained in detail and an information brochure given to consenting parents. A child was enrolled after written consent from at least one parent.

Each illness episode was assessed using a standard case report form. Main symptoms and signs were systematically reported using tick-boxes. In December 2007, pulse oximetry (Nellcor Pulse OxyMax N-20PA) was introduced at the Mugil study clinic and used systematically to assess peripheral arterial oxygen saturation of sick children. The normal values of SpO_2_, according to the WHO definitions, are between 97 and 100%, with a lower limit of 94% at sea level [19]; in the present study the health clinic staff were recommended to consider admission or referral of study participants if their peripheral arterial oxygen saturation dropped below 94%; moreover WHO recommendations were followed to administer oxygen if the SpO_2_ was < 90% [20]. In cases of history of fever in the previous 48 hours, or an axillary temperature > 37.5°C, a rapid diagnostic test (RDT) for malaria (ICT ComboH, South Africa) was performed and haemoglobin (Hb) levels measured using a portable Hemocue 201 machine (Angelholm, Sweden). Malaria treatment was based on RDT results. All illnesses were treated according to the standard treatment guidelines of PNG, based on Integrated Management of Childhood Illness (IMCI), except for malaria where an ad hoc study procedure with artemether/lumefantrine was used. Children were also assessed for danger signs and symptoms and referred for admission when necessary.

A severe illness was defined as an illness episode with potential life-threatening features as detailed in **Table 1**. At least one danger sign was needed to classify an episode as severe disease. A child with severe illness may not be admitted for any of the following reasons: parental refusal, personal judgment of health staff, or misidentification of danger signs & symptoms.

**Table 1:** Outcome definitions.

**Severe illness**	**At least one of the following danger signs:** ** - Respiratory distress ^1^.** ** - Inability to breastfeed or drink, or vomiting everything.** ** - Drowsy.** ** - Unable to sit.** ** - Neck stiffness.** ** - Fits.** ** - Severe dehydration.** ** - Severely pale.**
**Severe pneumonia**	Cough/difficulty breathing **and** Tachypnoea **2** **and** at least one of the following: - Severe respiratory distress ^1^. - Central cyanosis. - Inability to breastfeed or drink, or vomiting everything. - Convulsion, lethargy or unconsciousness.
**Pneumonia**	Cough/difficulty breathing **and** Tachypnoea/lower chest indrawing **and** no signs of severe pneumonia.
**Hospitalization**	Children that were hospitalized.

1 Defined by the presence of very severe chest indrawing and/or nasal flaring and/or grunting in young infants.

2 Defined as follows: respiratory rate more than or equal to 50/minute for children aged 2 to 12 months and more than or equal to 40/minute for those aged from 12 months to 5 years.

### Study staff

The clinical staff comprised 2 to 3 nurses and community health workers (CHW) specially trained for study procedures including completing case report forms and blood collection. Otherwise, care provided was very close to that usually performed in government health facilities, except there was no shortage of drugs or consumables.

### Study procedure

A clinical case was included if it fulfilled the following inclusion criteria:

1. Children aged between 3 and 27 months, enrolled in the IPTi study
2. Full clinical records (signs and symptoms) were available
3. Pulse oximetry was measured at time of visit

Children who visited the clinic in the prior 14 days were excluded from the study to avoid counting the same episode twice. Definitions of the measured outcomes are summarized in **Table 1**.

### Statistical analysis

We calculated the performance (sensitivity, specificity, positive and negative predictive values), unadjusted odds ratio (OR) and likelihood ratio (LR) of pulse oximetry peripheral arterial oxygen saturation with thresholds between 90% and 99% to identify severe illnesses from all cases, severe pneumonia from all pneumonias and hospitalizations from all cases.

Receiver operating characteristic (ROC) curves were drawn and areas under the curve (AUC) calculated for all three outcomes, global and stratified by age (≤ 12 months vs. > 12 months). AUCs in the two age groups were compared using AUC standard errors given in [21]. Optimal cut-off to predict outcome was obtained combining results of odds ratios and Youden Indexes.

## Results

**Fig 1:**
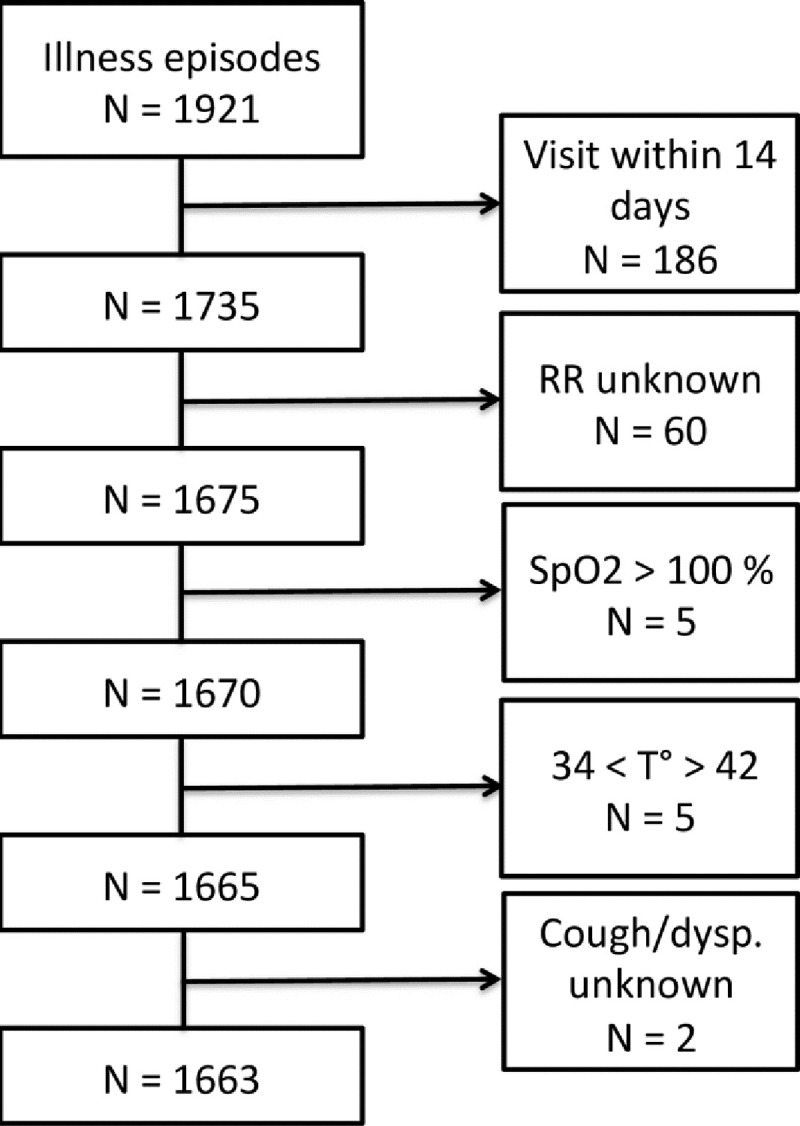
Study flowchart. Abbreviations: RR: Respiratory Rate, SpO_2_: peripheral arterial oxygen saturation measured with pulse oximetry, T°: Temperature.

**Fig 2:**
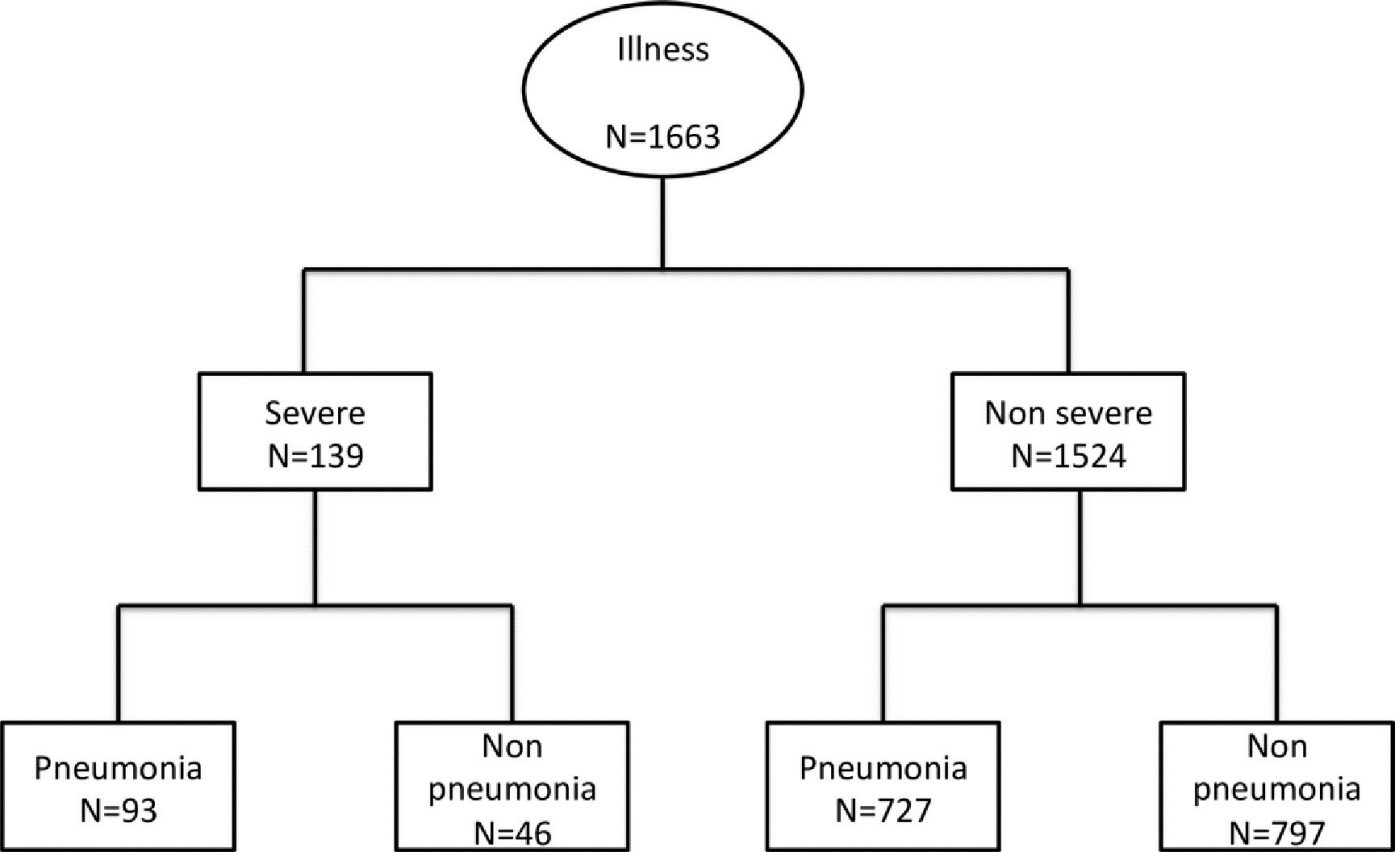
Classification tree of the illness episodes.

Between December 2007 and May 2010, 669 children visited the clinic, 327 (49%) were females and a total of 1921 illness episodes were recorded. We retained 1663 illness episodes that occurred in 643 children (312 females) fulfilling inclusion criteria. Details of excluded subjects are shown in **Figs 1**and **2**shows the illness classification tree.

No deaths were recorded. The general description of the cohort is shown in **Table 2.**

**Table 2:** General description of the cohort.

	All illness episodes
**Total**	**1663**
**Main signs, symptoms**	**N (%)**
Fever (>37.5°/hist. of Fever)	1590 (95.6)
Pallor	57 (3.4)
Difficulties in breathing	352 (21.2)
Cough	1316 (79.1)
Runny nose	947 (56.9)
Diarrhoea	472 (28.4)
Abdominal pain	63 (3.8)
Ear pain	8 (0.5)
Tachypnoea ^1^	970 (58.3)
Heart Rate ^2^	145 (19)
**Paraclinical findings**	**N (%)**
SpO2 = 100%	657 (39.5)
SpO2 = 99%	324 (19.5)
SpO2 = 98%	276 (16.6)
SpO2 = 97%	144 (8.7)
SpO2 = 96%	99 (6.0)
SpO2 = 95%	64 (3.8)
SpO2 = 94%	36 (2.2)
SpO2 = 93%	14 (0.8)
SpO2 = 92%	14 (0.8)
SpO2 = 91%	12 (0.7)
SpO2 ≤ 90%	23 (1.4)
Hb < 8 g/L	112 (6.7)
**Danger signs ^3^**	**N (%)**
Unable to eat	15 (0.9)
Vomit everything	7 (0.4)
Unconscious/drowsy	47 (2.8)
Respiratory distress ^4^	60 (3.6)
Fits	19 (1.1)
Inability to sit up	3 (0.2)
Neck stiffness	2 (0.1)
Severe dehydration	19 (1.1)
Severe pallor	5 (0.3)

^**1**^: defined as ≥ 50 breaths/min for children aged 2–11 months and ≥ 40 breaths/min for children ≥12 months.

^**2**^: data are mean (standard deviation).

^**3**^: more than one danger sign is possible.

^**4**^: Defined by the presence of severe chest indrawing and/or nasal flaring and/or grunting in young infants.

The mean value of SpO_2_ was 96.9% (SD 3.7%) in episodes presenting ≥1 danger sign (n = 139) and 98.4% (SD: 2.2%) in those without danger sign (n = 1524) (p<0.001). It was 96.6% (SD: 3.9%) and 98.4% (SD 2.2%) in episodes presenting criteria of severe pneumonia (n = 93) and in those with non-severe pneumonia (n = 727), respectively (p<0.001). It was 96.0% (SD 4.0%) and 98.1% (SD 2%) in children hospitalized (n = 84) and those non-hospitalized (n = 1579) respectively (p<0.001).

**Fig 3:**
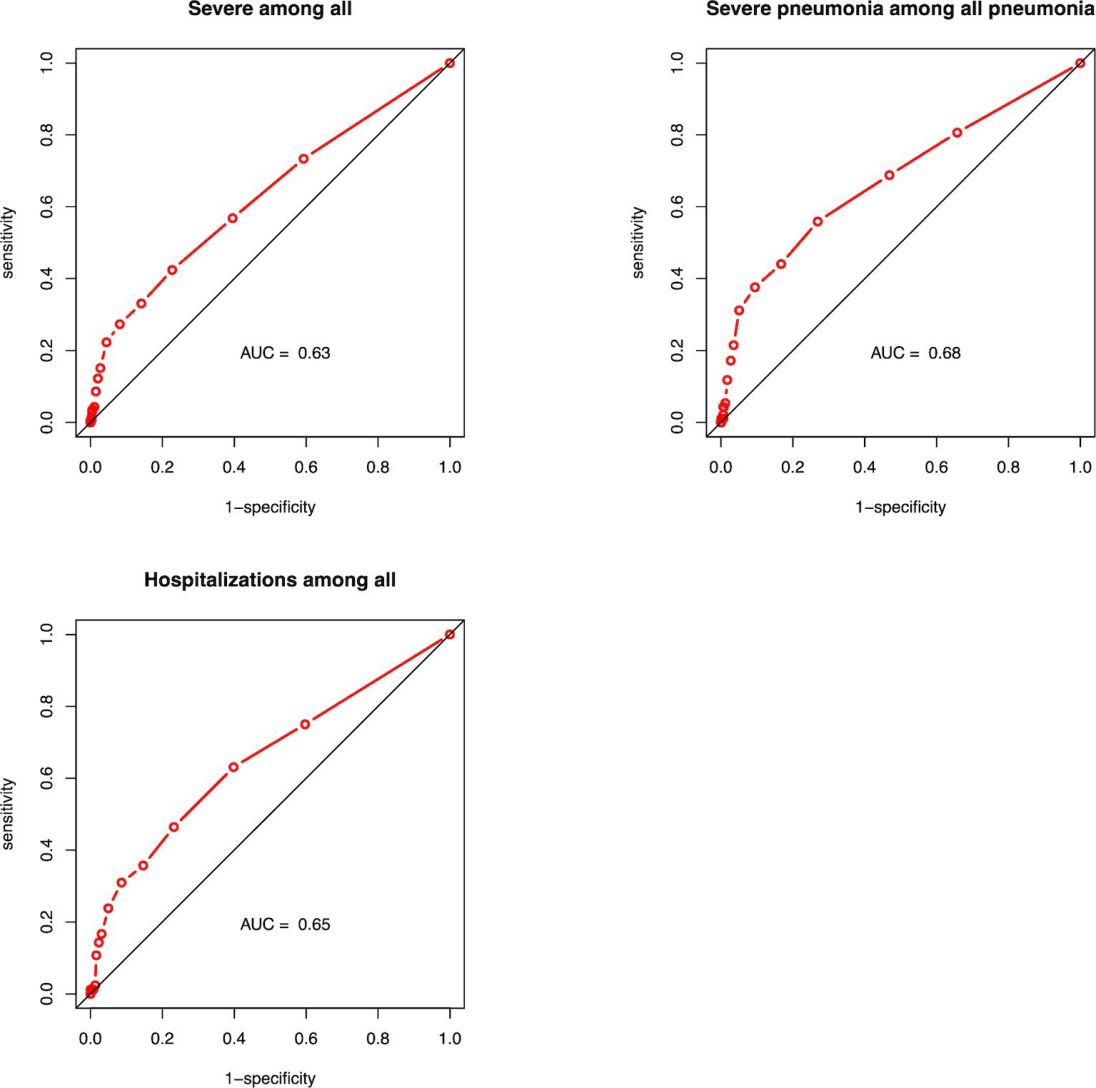
ROC curves to predict: Severe diseases among all illness episodes, severe pneumonia among all pneumonias episodes and hospitalizations among all illness episodes.

**Fig 4:**
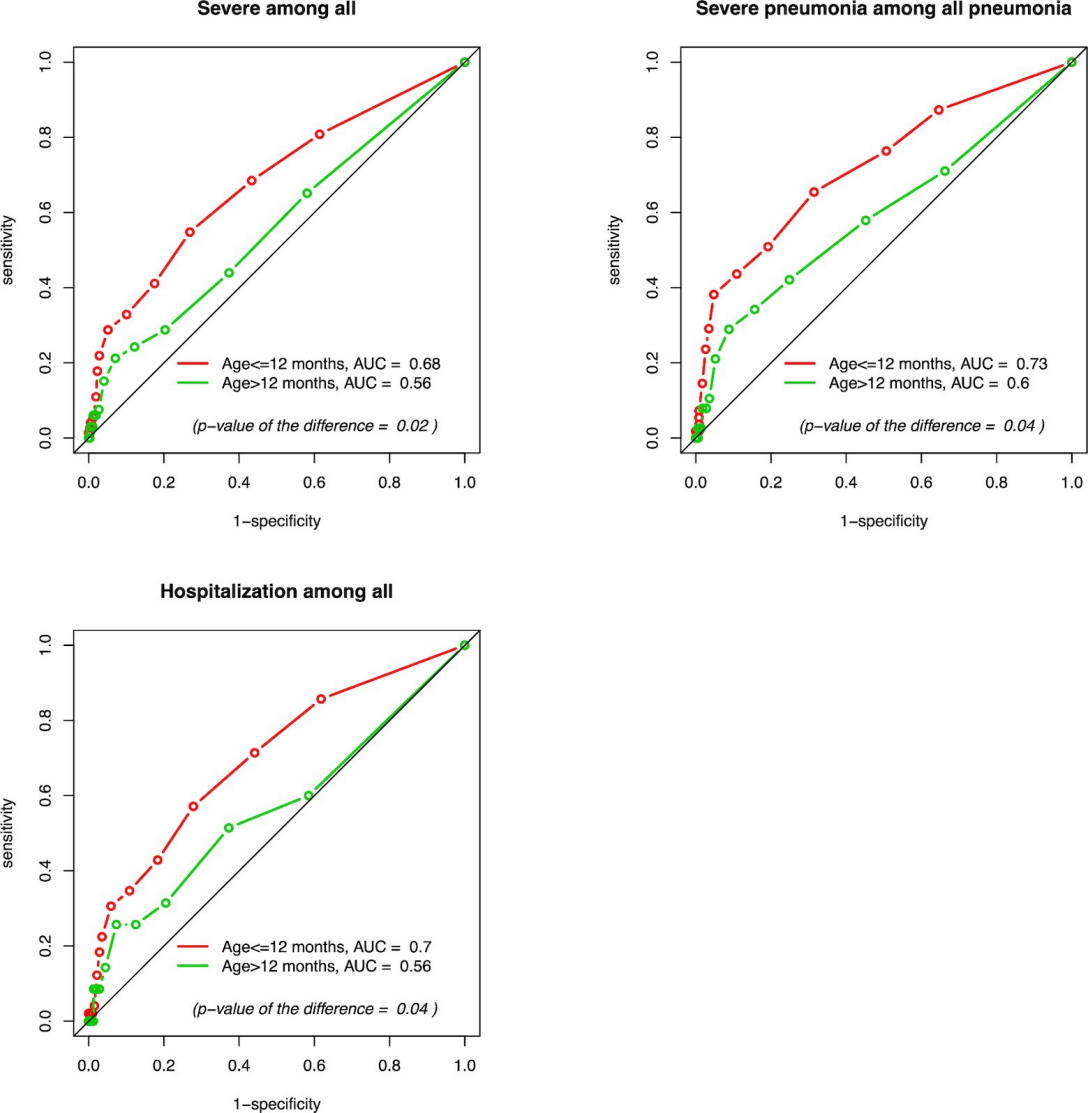
ROC curves stratified by age to predict: Severe diseases among all illness episodes, severe pneumonia among all pneumonias episodes and hospitalizations among all illness episodes.

Among 1663 illness episodes, 139 were classified as severe illness. Using SpO_2_ thresholds between 99% and 90%, sensitivity of pulse oximetry to predict severe illness among all illnesses ranged from 73 to 4%, specificity from 41 to 99%, positive predictive value (PPV) from 10 to 26% and negative predictive value (NPV) from 94 to 92%. The AUC for SpO_2_ to predict severe illness was 0.63 (**Fig 3**). When stratified by age, the AUC was higher for children ≤12-months compared to children >12-months (0.68 and 0.56, respectively; p = 0.02) (**Fig 4**).

Among 820 pneumonia episodes, 93 episodes were classified as severe pneumonia. Using SpO_2_ thresholds between 99% and 90%, sensitivity of pulse oximetry to predict severe pneumonia among all pneumonia ranged from 81 to 5%, specificity from 34 to 98%, PPV from 14 to 46% and NPV from 93 to 89%. The AUC for SpO_2_ to predict severe pneumonia was 0.68 (**Fig 3**). When stratified by age, the AUC for children ≤12-months was 0.73 while it was 0.60 for children >12-months (p = 0.04) (**Fig 4**).

Among 1663 illness episodes, 84 episodes ended with hospitalization. For Using SpO_2_ thresholds between 99% and 90%, sensitivity of pulse oximetry in predicting hospitalizations among all illnesses ranged from 75 to 2%, specificity from 40 to 99%, PPV from 6 to 26% and NPV from 97 to 95%. The AUC for SpO_2_ to predict hospitalization was 0.65 (**Fig 3**). When stratified by age, the AUC was higher for children ≤12-months compared to children >12-months (0.70 and 0.56, respectively; p = 0.04) (**Fig 4**).

**Fig 5:**
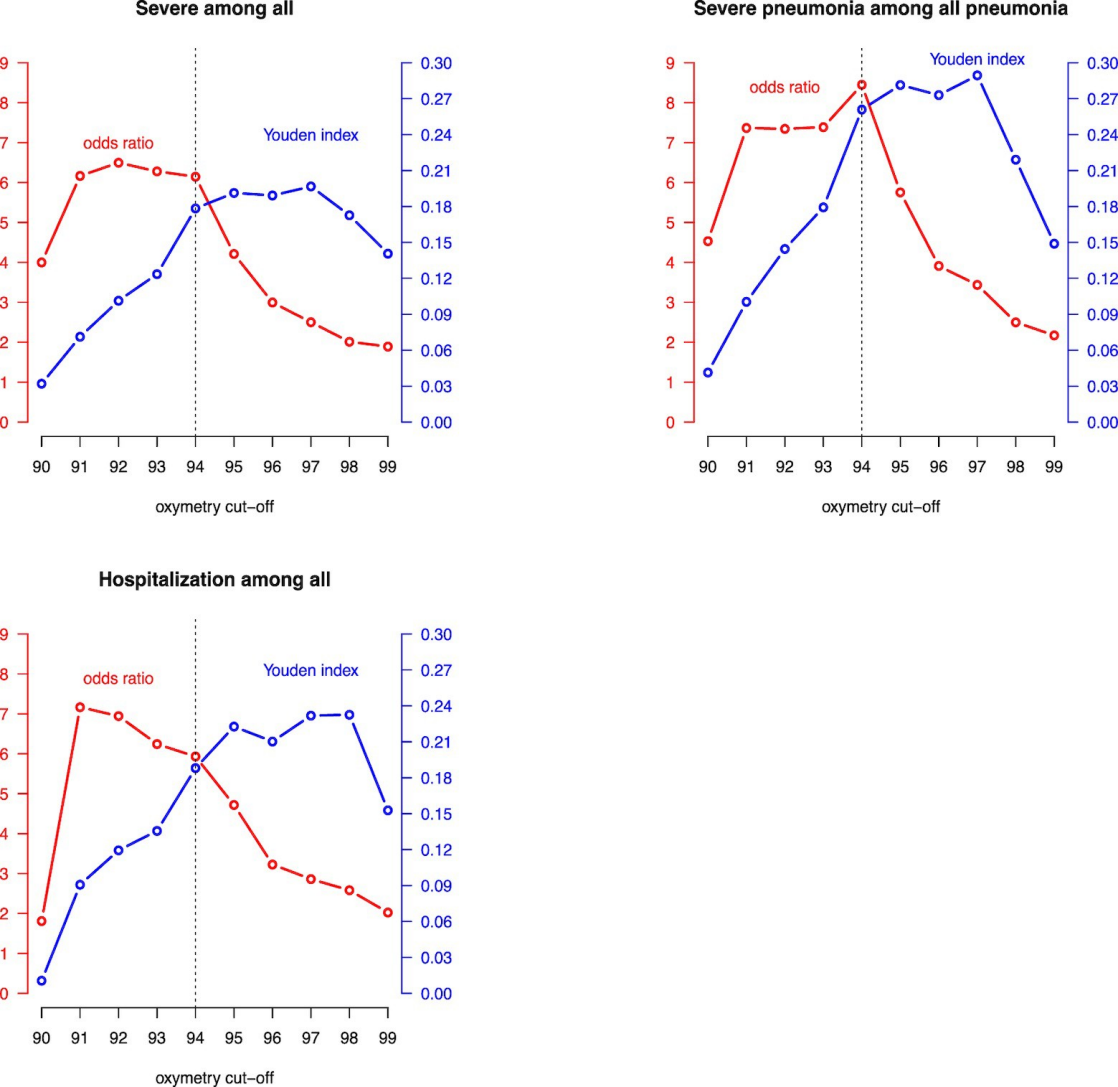
ORs and Youden Indexes for different cut-offs of SpO_2_.

The ORs and Youden Indexes according to SpO_2_ threshold for prediction of the three outcomes (severe illness, severe pneumonia and hospitalization) are given in **Fig 5**.

## Discussion

Only a few studies have explored the utility of pulse oximetry to identify severely ill children in a remote day-to-day pediatric outpatient clinic. It was found that a low SpO_2_ correlates with severity of illness, regardless of etiology. As expected, the association with severe illnesses was stronger when the SpO_2_ was lower and the association was also stronger if severe illnesses included respiratory symptoms. An AUC of 0.60 for severe pneumonia represents a fair performance although we expected better performance. It suggests that SpO2 values alone have only a limited discriminative power. As shown in other studies, a combination of clinical signs and pulse oximetry values could lead to better performance [15,16].

We observed better performance of pulse oximetry in younger children, independent of the outcome (AUC of 0.68 for severe illness among all illness episodes and 0.73 for severe pneumonia among all pneumonia). This observation is particularly interesting because identification of severe illness is more complicated in younger children and pulse oximetry could be therefore a valuable tool in this sub-group. Reasons for this are unclear, but it is possible that younger children have more immature lung function and thus even a mild disease might lead quickly to a decrease in SpO_2_.

Pulse oximetry showed high NPVs, constantly above 90% that are comparable or even better than those found in the meta-analysis of Zhang & al., suggesting that pulse oximetry can be a useful additional tool to reasonably exclude severe illnesses in children presenting at an outpatient clinic in a developing country, a lower prevalence setting compared to an emergency department.

WHO recommends oxygen administration to children with severe pneumonia if the SpO_2_ falls under 90% [20]. The British Thoracic Society guidelines for the management of community-acquired pneumonia (CAP) in children highlights that there is not a single validated severity score to guide the decision for hospitalization. In an emergency care-based study that evaluated which children were at risk of a severe infection using vital signs, a threshold of a SpO_2_ <94% was proposed. For unclear reasons a threshold for hospitalization of SpO_2_ < 92% was finally proposed [22]. A systematic review and meta-analysis that evaluated the association between hypoxemia and mortality in children with acute lower respiratory infection in mid- and low-income countries, showed that children with SpO2 values of less than 90% and 92% had an increased risk of mortality, but the OR between the two cut-offs showed no significant difference [23]. A recent systematic review proposes giving oxygen to all children presenting with WHO-defined emergency signs with a SpO_2_ < 95%, but the threshold should be weighted with the oxygen-availability supplies [24]. In the present study, it seems appropriate to consider referral for admission or further care if the SpO_2_ is ≤94% [25]. **Fig 5**shows that a cut-off of SpO_2_ ≤ 94% seems the best compromise, with high OR and Youden Index that are only slightly smaller than their maximum values.

Implications for routine practice:

Often in ambulatory settings, the paramedical staff is the first health staff to encounter sick children with limited training in recognizing clinical signs of hypoxemia that makes it difficult to identify severe illnesses and severe acute respiratory infections. Thus, the introduction of a simple objective and reproducible measure of hypoxemia such as pulse oximetry would be welcome. The use of pulse oximetry is extremely easy and affordable making it the tool of choice for large screenings in relatively low-prevalence rural settings (in our study, less than 10% of illness could be considered clinically severe). Moreover, a better evaluation of the peripheral arterial oxygen saturation could lead to a more rational use of oxygen therapy.

In addition to evaluating the performance of pulse oximetry as a diagnostic tool, this study explored the feasibility of implementing it, which indicated that pulse oximetry can be confidently used in day-to-day practice by staff with limited clinical expertise. Its implementation in the frame of existing guidelines such as the IMCI, therefore seems feasible with the potential to improve substantially the management of severe pneumonia and more generally severe illnesses. As a recent systematic review highlights [26], more prospective studies are needed to assess how pulse oximetry could impact on illness outcome.

## Conclusion

This study provided some evidence for the usefulness of measuring oxygen saturation by pulse oximetry in children in resource-poor outpatient clinics in improving the identification of children with severe pneumonia and more generally severe illnesses, especially in children under one year. A SpO_2_ of 94% appeared to be a reasonable threshold for considering further examination and observation. Furthermore, this study showed that it is feasible to implement such a device in the context of an outpatient clinic with limited resources. Due to its ease of use and affordability, policy makers should consider introducing it into the IMCI recommendations.

## Supporting information

S1 Table Danger signs among all illness episodes. Se: sensitivity, Sp: specificity, PPV: positive predictive value, NPV: negative predictive value, LR +: positive likelihood ratio, LR-: negative likelihood ratio, OR: odd ratio.
(TIF)

S2 Table Severe pneumonia among all pneumonia episodes. Se: sensitivity, Sp: specificity, PPV: positive predictive value, NPV: negative predictive value, LR +: positive likelihood ratio, LR-: negative likelihood ratio, OR: odd ratio.
(TIF)

S3 Table Hospitalization among all illness episodes. Se: sensitivity, Sp: specificity, PPV: positive predictive value, NPV: negative predictive value, LR +: positive likelihood ratio, LR-: negative likelihood ratio, OR: odd ratio.
(TIF)
