# Supplementary figures and images for: Retrospective study on the usefulness of pulse oximetry for the identification of young children with severe illnesses and severe pneumonia in a rural outpatient clinic of Papua New Guinea
Source: PLoS One. 2019 Apr 15;14(4):e0213937. doi: 10.1371/journal.pone.0213937 (PMC6464326; doi:10.1371/journal.pone.0213937)

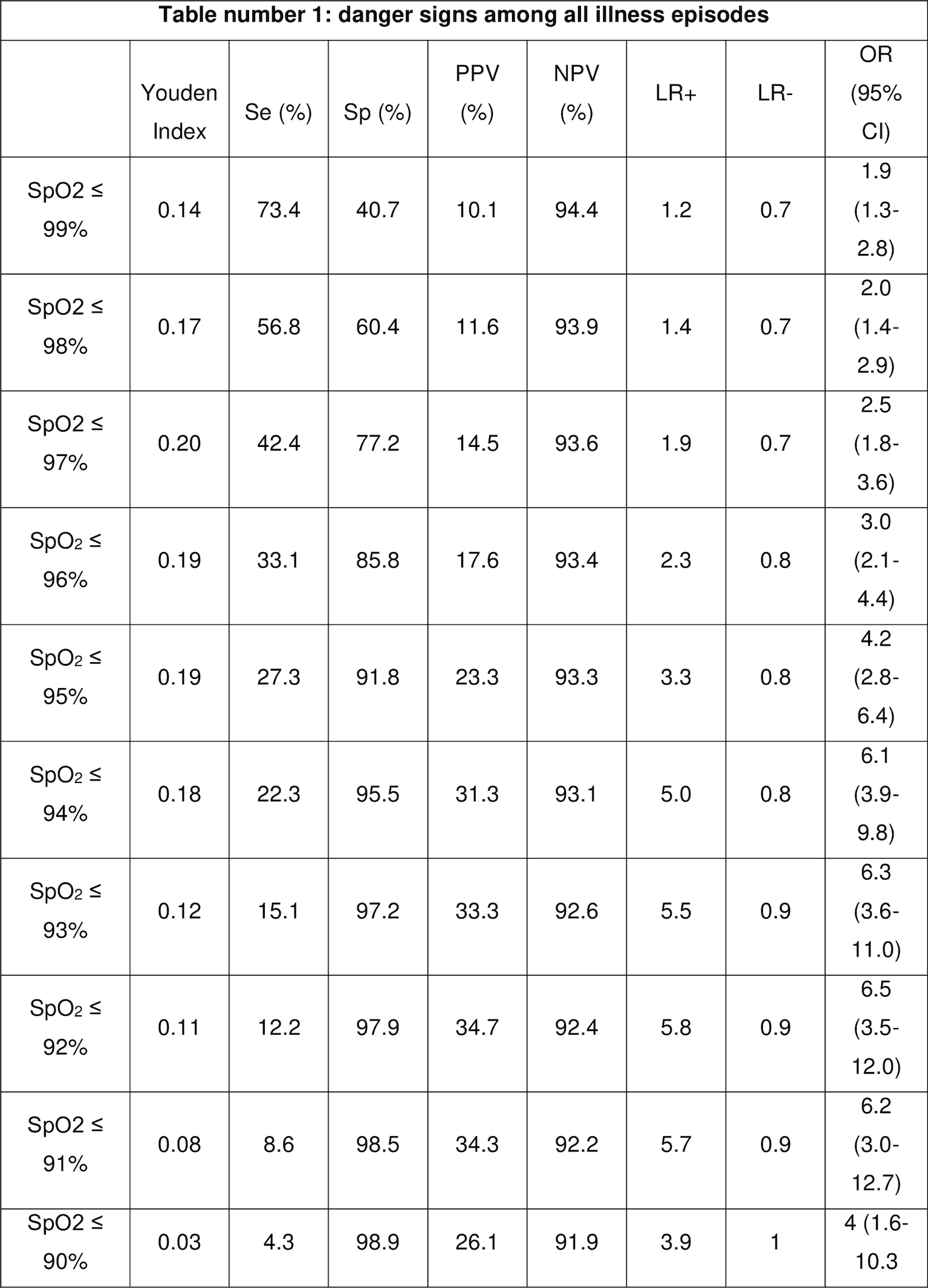

Supplement: S1 Table — Se: sensitivity, Sp: specificity, PPV: positive predictive value, NPV: negative predictive value, LR +: positive likelihood ratio, LR-: negative likelihood ratio, OR: odd ratio. (TIF) [file pone.0213937.s001.tif]

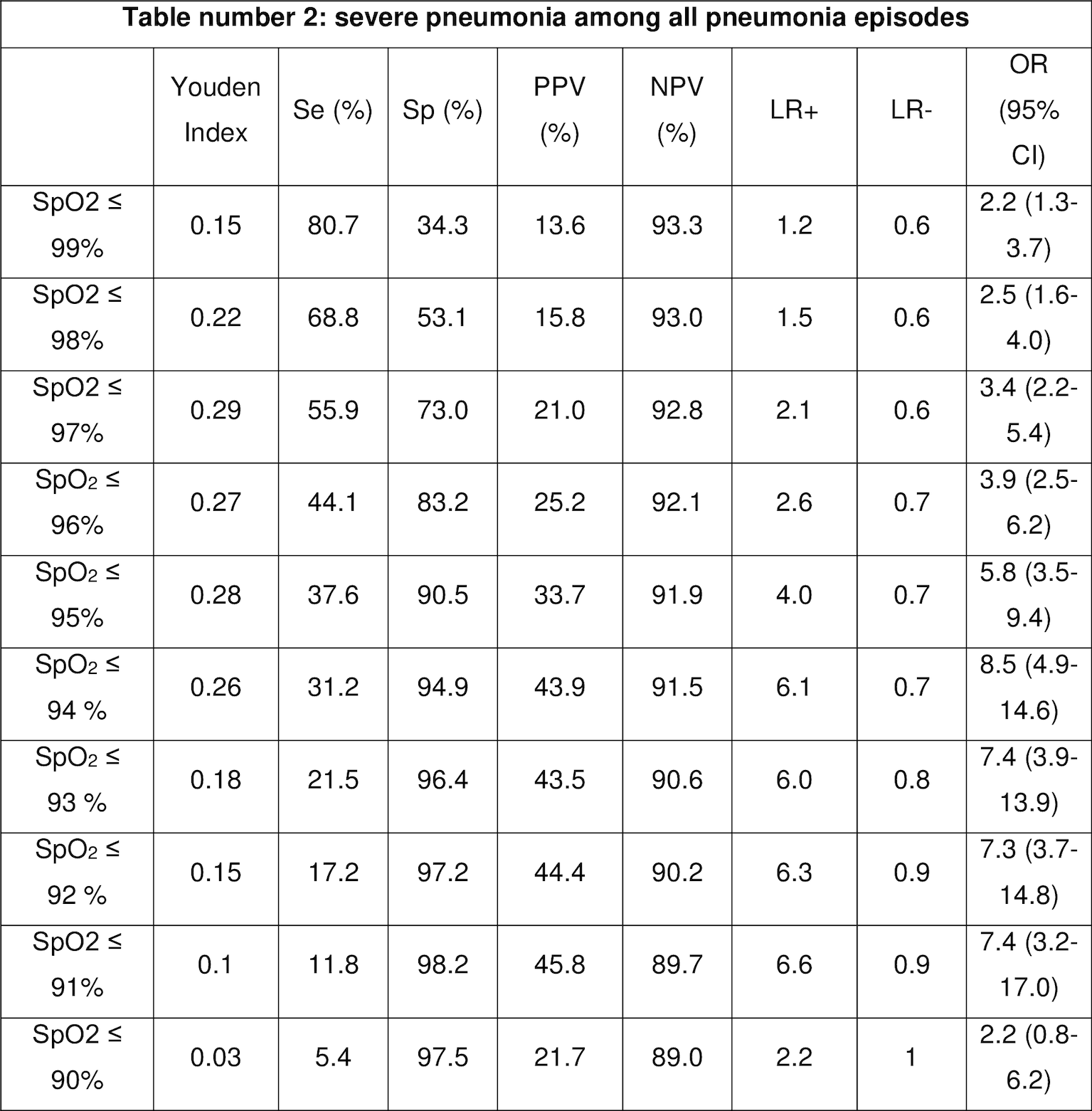

Supplement: S2 Table — Se: sensitivity, Sp: specificity, PPV: positive predictive value, NPV: negative predictive value, LR +: positive likelihood ratio, LR-: negative likelihood ratio, OR: odd ratio. (TIF) [file pone.0213937.s002.tif]

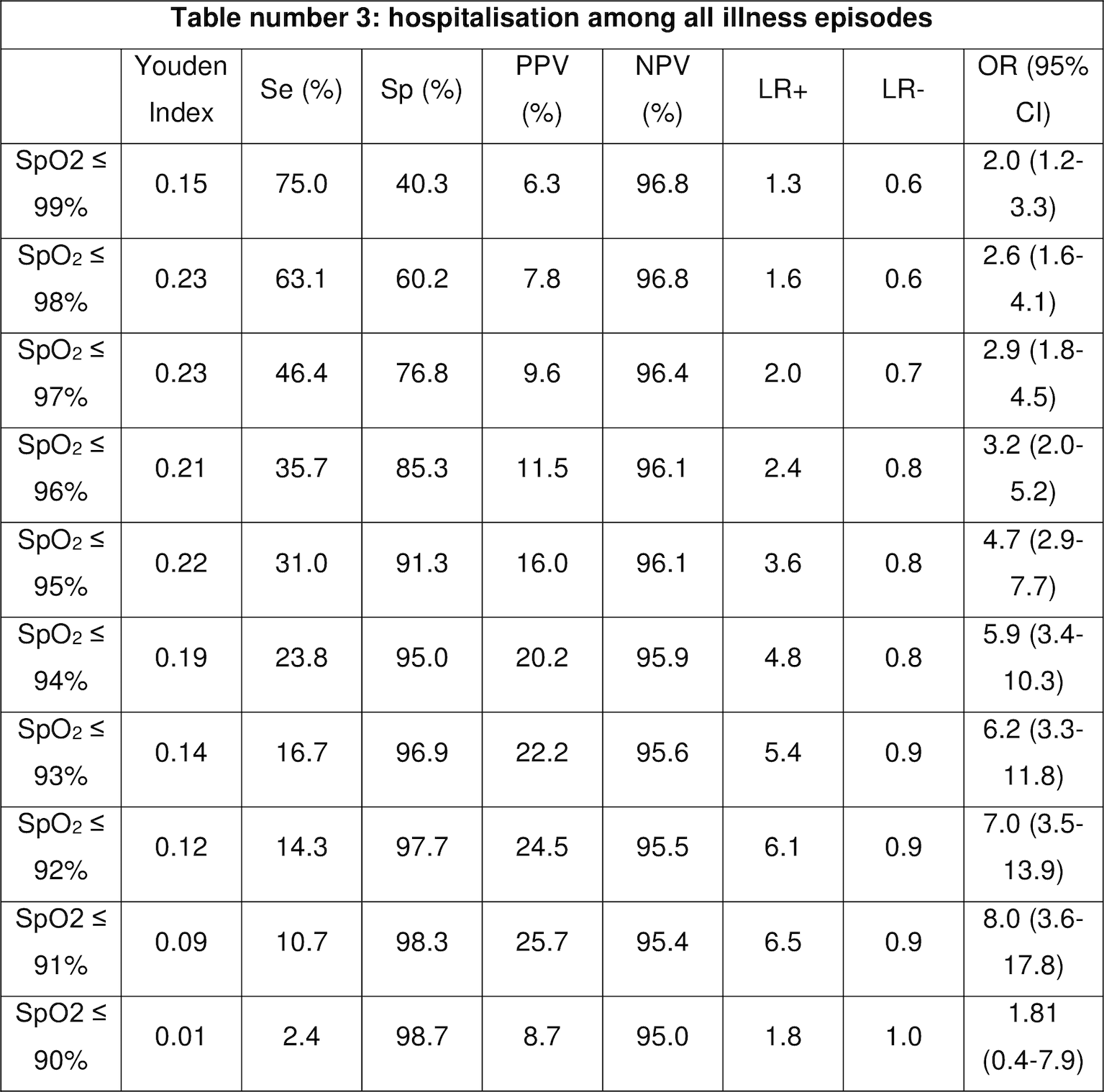

Supplement: S3 Table — Se: sensitivity, Sp: specificity, PPV: positive predictive value, NPV: negative predictive value, LR +: positive likelihood ratio, LR-: negative likelihood ratio, OR: odd ratio. (TIF) [file pone.0213937.s003.tif]
